# NOX2 amplifies acetaldehyde-mediated cardiomyocyte mitochondrial dysfunction in alcoholic cardiomyopathy

**DOI:** 10.1038/srep32554

**Published:** 2016-09-14

**Authors:** Moritz Brandt, Venkata Garlapati, Matthias Oelze, Efthymios Sotiriou, Maike Knorr, Swenja Kröller-Schön, Sabine Kossmann, Tanja Schönfelder, Henning Morawietz, Eberhard Schulz, Heinz-Peter Schultheiss, Andreas Daiber, Thomas Münzel, Philip Wenzel

**Affiliations:** 1 Cardiology 1, Center for Cardiology, University Medical Center Mainz, Langenbeckstr. 1, 55131 Mainz, Germany; 2 Center for Thrombosis and Hemostasis Mainz, University Medical Center Mainz, Langenbeckstr. 1, 55131 Mainz, Germany; 3 German Center for Cardiovascular Research (DZHK), partner site Rhine Main, University Medical Center Mainz, Langenbeckstr. 1, 55131 Mainz, Germany; 4 Division of Vascular Endothelium and Microcirculation, Department of Medicine III, University Hospital Carl Gustav Carus, Technische Universität Dresden, Fetscherstr. 74, 01307 Dresden, Germany; 5 Institut für kardiale Diagnostik und Therapie, Moltkestrasse 31, 12203 Berlin, Germany

## Abstract

Alcoholic cardiomyopathy (ACM) resulting from excess alcohol consumption is an important cause of heart failure (HF). Although it is assumed that the cardiotoxicity of the ethanol (EtOH)-metabolite acetaldehyde (ACA) is central for its development and progression, the exact mechanisms remain obscure. Murine cardiomyocytes (CMs) exposed to ACA or EtOH showed increased superoxide (O_2_^•−)^ levels and decreased mitochondrial polarization, both being normalized by NADPH oxidase (NOX) inhibition. C57BL/6 mice and mice deficient for the ACA-degrading enzyme mitochondrial aldehyde dehydrogenase (ALDH-2^−/−^) were fed a 2% EtOH diet for 5 weeks creating an ACA-overload. 2% EtOH-fed ALDH-2^−/−^ mice exhibited a decreased cardiac function, increased heart-to-body and lung-to-body weight ratios, increased cardiac levels of the lipid peroxidation product malondialdehyde (MDA) as well as increased NOX activity and NOX2/glycoprotein 91^phox^ (NOX2/gp91^phox^) subunit expression compared to 2% EtOH-fed C57BL/6 mice. Echocardiography revealed that ALDH-2^−/−^/gp91^phox−/−^ mice were protected from ACA-overload-induced HF after 5 weeks of 2% EtOH-diet, demonstrating that NOX2-derived O_2_^•−^ contributes to the development of ACM. Translated to human pathophysiology, we found increased gp91^phox^ expression in endomyocardial biopsies of ACM patients. In conclusion, ACM is promoted by ACA-driven mitochondrial dysfunction and can be improved by ablation of NOX2/gp91^phox^. NOX2/gp91^phox^ therefore might be a potential pharmacological target to treat ACM.

Heart failure (HF) is a major public health problem with a prevalence of more than 23 million worldwide. Alcoholic cardiomyopathy (ACM) is a major contributor to the emerging epidemic of HF, accounting for about 3.8% of all cardiomyopathy cases^1^ and 21–36% of nonischemic-dilated cardiomyopathy cases^2^,^3^. ACM is characterized by an increase in left ventricular mass, dilation of the ventricles, wall thinning and ventricular dysfunction, with all these changes present in the absence of any other cause of intrinsic or extrinsic cardiomyopathy^1^. Arrhythmias and electrocardiographic abnormalities may also be present in ACM patients with hemodynamically relevant alcohol-induced sinus bradycardia leading to recurrent syncope as the most significant bradyarrhythmia^2^.

The amount and duration of per day consumption of ethanol (EtOH) sufficient to cause structural and functional myocardial alterations has been reported to start at as little as 80 g per day over 5 years^1^,^4^; a finding that has recently been paralleled by a large meta-analysis, which saw a lack of protective associations among most age-specific and sex-specific alcohol use categories compared with non-alcohol consumers and thereby challenged the long standing dogma of a protective effect of moderate alcohol consumption on cardiovascular diseases^5^.

Acetaldehyde (ACA) is considered the main culprit in the pathogenesis of ACM due to its high biochemical reactivity^6^ and its ability to concentrate in the myocardium^7^. Cytosolic alcohol dehydrogenase (ADH) forms ACA via partial oxidation of EtOH, while ACA is further oxidized to acetate by the mitochondrial aldehyde dehydrogenase (ALDH-2). Higher levels of ACA in drinkers with a genetic variation resulting in an overactive ADH (ADH 1C*1) were shown to be associated with an increased risk of several alcohol-related cancers^8^. In a mouse model of chronic alcohol administration, increased myocardial levels of ACA accelerated the development of cardiomyopathy in cardiomyocyte-specific ADH-overexpressing mice compared to control mice^9^,^10^,^11^. Of note, even short-term exposure of CMs to ACA was sufficient to compromise cellular function in these cells^6^. Vice versa, EtOH severely compromised myocardial and cardiomyocyte function in wild-type FVB mice, whereas overexpression of ALDH-2 in transgenic mice abolished the detrimental effects of the acute^12^ and chronic EtOH exposure^13^. These observations are also in good accordance with more recent findings that ALDH-2 is a cardioprotective enzyme that confers protection from ischemic damage during myocardial infarction and a significant decrease in the infarct area^14^,^15^.

Similar to the overactive ADH 1C*1-isoform, the ALDH-2 mutant allele (ALDH-2*2) with a gene product that has no or very low ACA-oxidizing capacity^16^ leads to the accumulation of ACA. After alcohol consumption, carriers of this mutant allele experience symptoms caused by increased ACA toxicity often associated with the flushing syndrome^17^. It remains elusive, however, whether the cardiotoxicity of EtOH and ACA additionally involves reactive oxygen species (ROS). Interestingly, EtOH and ACA-induced mitochondrial O_2_^•−^ production in mice with cardiac-specific overexpression of ADH resulted in activation of the mitochondrial death pathway and myocardial apoptosis^10^. Furthermore, EtOH metabolism itself can be a source of O_2_^•−^. In addition to ADH and ALDH-2, the cytochrome P450 (CYP) oxidase 2E1 is involved in EtOH metabolism^18^. Due to its induction by EtOH^19^, its importance might increase under conditions of chronic EtOH uptake. EtOH oxidation to ACA by CYP 2E1 involves the generation of hydroxyl radicals via the Fenton/Haber-Weiss reaction^20^. However, ROS-generating enzymes like NOXs, specifically NOX2, activated by ACA via a transcriptional process might even outweigh the direct ROS production from EtOH breakdown^21^. This hypothesis of NOX as a factor in EtOH/ACA toxicity coincides with a large body of evidence that NOXs are involved in the pathogenesis of HF^22^,^23^. NOX2 has been shown to drive HF as it contributes to cardiomyocyte hypertrophy, apoptosis and interstitial fibrosis^24^,^25^. Hence, we investigated the role of myocardial ROS production and NOX function in the pathogenesis of ACM by inducing ACA-overload in EtOH fed ALDH-2^−/−^ mice and by generating ALDH-2^−/−^/gp91^phox−/−^-mice.

## Results

### ACA-toxicity leads to decompensated HF with cardiomegaly, pulmonary edema and cardiac fibrosis in ALDH-2^−/−^ mice.

**Figure 1:**
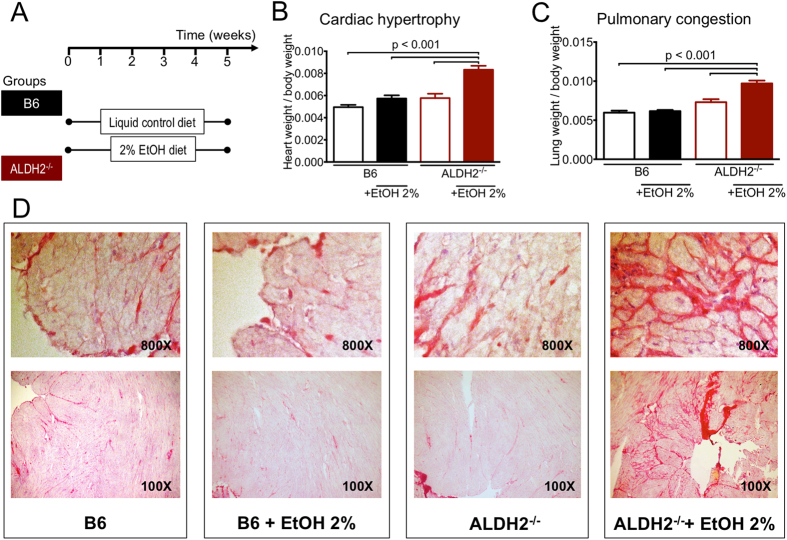
ACA-overload leads to cardiac enlargement myocardial fibrosis. (**A**) Scheme depicting the treatment of C57BL/6 and ALDH-2^−/−^ mice with 2% EtOH diet and liquid control diet. (**B**) Heart weight/body weight-ratios and (**C**) Lung weight/body weight-ratios in C57BL/6 and ALDH-2^−/−^ animals fed a 2% EtOH diet vs. liquid control diet. Data are presented as the mean ± SEM from n** = **12–14 animals per group. (**D**) Myocardial sections stained with Sirius-red from C57BL/6 and ALDH-2^−/−^ animals fed a 2% EtOH diet vs. liquid control diet.

When fed a 2% EtOH-Lieber-deCarli diet (EtOH diet) for 5 weeks (for protocol see Fig. 1A), ALDH-2^−/−^ mice had increased heart-to-body weight and lung-to-body weight ratios compared to ALDH-2^−/−^ mice fed the control diet or compared to C57BL/6 mice fed either diet (Fig. 1B,C), indicative of the onset of cardiac enlargement and pulmonary congestion in these animals.

Staining of cardiac sections with Picro-Sirius red yielded an intensive staining pattern throughout the entire myocardium in ALDH-2^−/−^ mice fed the 2% EtOH diet, which was not visible in ALDH-2^−/−^-mice fed the control diet or C57BL/6 mice fed either diet, pointing towards an increased collagen deposition as part of a fibrosis in the myocardium of these animals (Fig. 1D).

These findings reveal that in ALDH2^−/−^ mice, a 2% EtOH-Lieber-deCarli diet results in a HF phenotype with cardiomegaly, pulmonary edema and cardiac fibrosis.

### NOX predominantly contributes to the ROS-associated myocardial damage in a mouse model of ACA toxicity.

**Figure 2:**
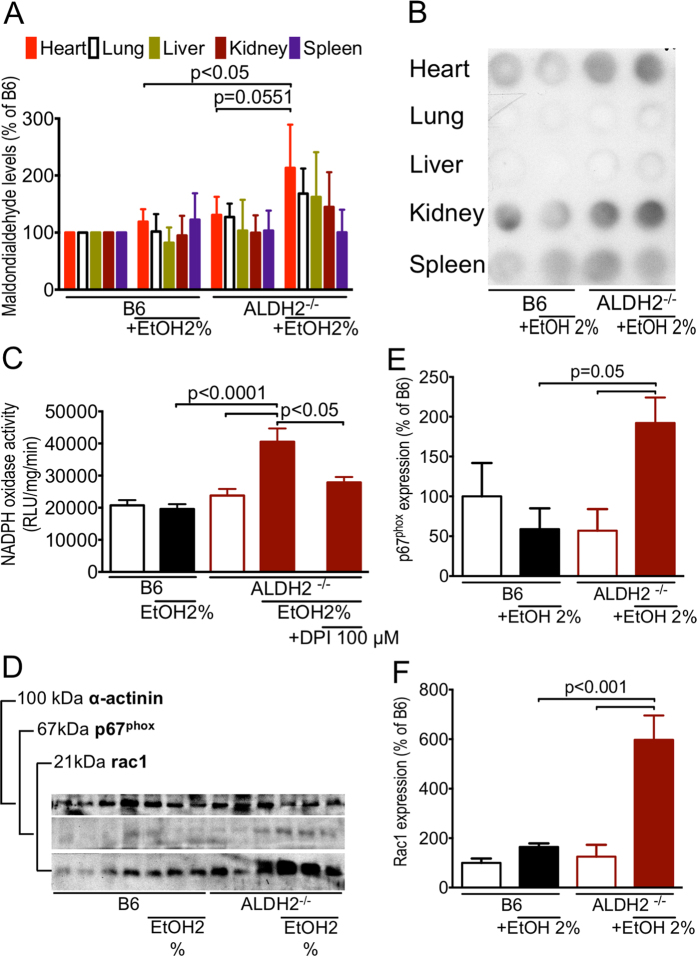
Increased myocardial ROS in ALDH-2^−/−^ mice fed a 2% alcoholic diet. (**A**) Malondialdehyde levels in internal organs as determined by dot blot analysis of tissue homogenates. Data are expressed as mean ± SEM from n** = **4 per group. (**B**) Representative original dot blot; (**C**) NOX activity in myocardial membrane fractions determined by lucigenin-derived chemiluminescence. Data are presented as mean ± SEM from 18–20 measurements of n** = **5 animals per group. (**D**) Representative original Western blot. (**D**) p67^phox^ protein expression and (**F**) Rac1 protein expression determined by Western blot. Data presented as mean ± SEM from n** = **3–4 animals per group.

In order to investigate the underlying mechanism responsible for the HF phenotype resulting from the ACA-overload, we measured malondialdehyde (MDA) levels in tissue homogenates from ALDH-2^−/−^ mice and C57BL/6 mice (subjected to both diets) by dot-blot analysis. The myocardial MDA levels in EtOH-fed ALDH-2^−/−^ mice were the highest among all organs and significantly higher than the corresponding levels in EtOH-fed C57BL/6 animals and control-fed ALDH-2^−/−^ mice (Fig. 2A,B).

Cardiac membrane fractions from ALDH-2^−/−^ mice fed the EtOH diet produced a significantly higher O_2_^•−^ signal than fractions from control-fed ALDH-2^−/−^ mice or from C57BL/6 mice fed either diet. Importantly, co-incubation with the NOX inhibitor diphenyleneiodonium (DPI) almost normalized the significantly increased chemiluminescence signal in myocardial membrane fractions from EtOH-fed ALDH-2^−/−^ mice (Fig. 2C).

Western blot analysis revealed a strong trend for increased expression of the NOX isoform NOX2 activator subunit p67^phox^ (p** = **0.0539 vs. ALDH-2^−/−^ fed a control diet) and a significantly increased expression of the ubiquitously distributed NOX1 and NOX2 activator of the Rho-family, the GTPase Rac1 (Fig. 2D–F) in myocardial membrane fractions from EtOH-fed ALDH-2^−/−^ mice compared to control-fed ALDH-2^−/−^-mice or to C57BL/6 mice fed either diet.

Taken together, these findings point towards ROS mediated myocardial damage underlying the HF phenotype with cardiomegaly and cardiac fibrosis, and further points towards NOXs as major contributors to the detrimental ROS burden in our animal model of ACA toxicity.

### Exposure of isolated CMs to EtOH or ACA induces upregulation NOX2 subunits and NOX2 activator proteins of the Rho family.

**Figure 3:**
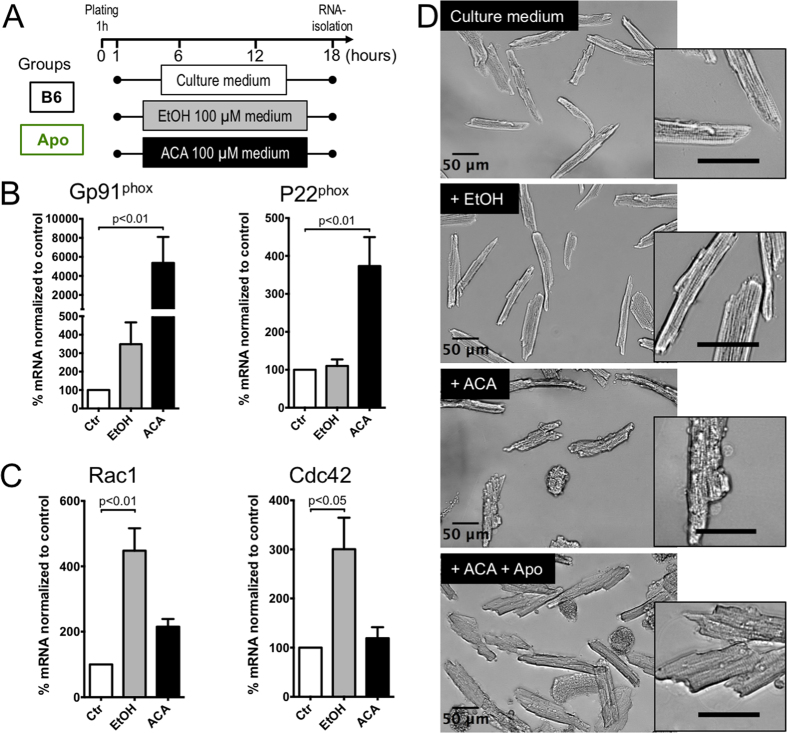
Exposure of isolated CMs to EtOH or ACA induces upregulation of NOX. (**A**) Scheme depicting incubation of isolated adult CMs with EtOH (100 μM) or ACA (100 μM). (**B**) Expression of NOX2 subunit gp91^phox^ and p22^phox^. (**C**) Expression of Rac GTPase Rac1 and of the Rho-family GTP binding protein cell division cycle 42 (CDC42). (**D**) Brightfield images of live mouse adult CMs after incubation in media supplemented with EtOH or ACA with or without apocynin, the scale bars were kept at 50 μM. (**B**,**C**) Data presented as the mean ± SEM.

To further investigate the effects of EtOH and ACA on the CM phenotype and to identify the NOX isoform responsible for the detrimental ROS burden in ACM, purified murine adult CMs were cultured in media containing EtOH or ACA (each 100 μM, equivalent to a blood concentration of 0.6% w/w) (for the protocol see Fig. 3A).

The mRNA-expression of NOX2/gp91^phox^ and the NOX2 subunit p22^phox^ (a critical factor for stability and interaction of NOX2/gp91^phox^ and its cytosolic subunits) were significantly increased in CMs in the presence of ACA (Fig. 3B), while the expression of NOX activator Rac1 and the cytosolic subunit p47^phox^, which is crucial for NOX2 activation^26^, was significantly increased after culture in the presence of EtOH (Figs 3C and S3). Interestingly, the cell cycle regulator protein cell division cycle 42 (CDC42), which has been shown to mediate the NOX2-activation responsible for oxidative damage induced by arsenic in endothelial cells^27^ and by ethanol in neurons^28^, was significantly upregulated on the mRNA level in the presence of EtOH (Fig. 3C). Of note, the level of NOX4 mRNA did not change significantly in the presence of EtOH (Fig. S3).

As observed in brightfield phase contrast microscopy of live CMs, the detrimental effects of ACA were observable after culture for 18 h: While control-cultured CMs remained in the typical rod-shape, which enables CM function in the heart with clear boundaries and crisp sarcomeric structure at high magnification, ACA-cultured CMs degenerated to a round “meatball” shape indicating a terminal hypercontraction or exhibited cell blebbing and a total loss of the sarcomeric ultrastructure. Further addition of apocynin improved CM morphology and maintained rod-shape and intact ultrastructure (Fig. 3D).

Taken together, these findings reveal that the significantly increased ROS production underlying the HF phenotype is paralleled by significantly increased expression of NOX2 subunits and significantly increased expression of NOX2 activators in isolated CM cultured in the presence of EtOH or ACA.

### Pharmacologic inhibition or genetic ablation of NOX reduces mitochondrial superoxide production in isolated CMs cultured in the presence of EtOH and ACA.

**Figure 4:**
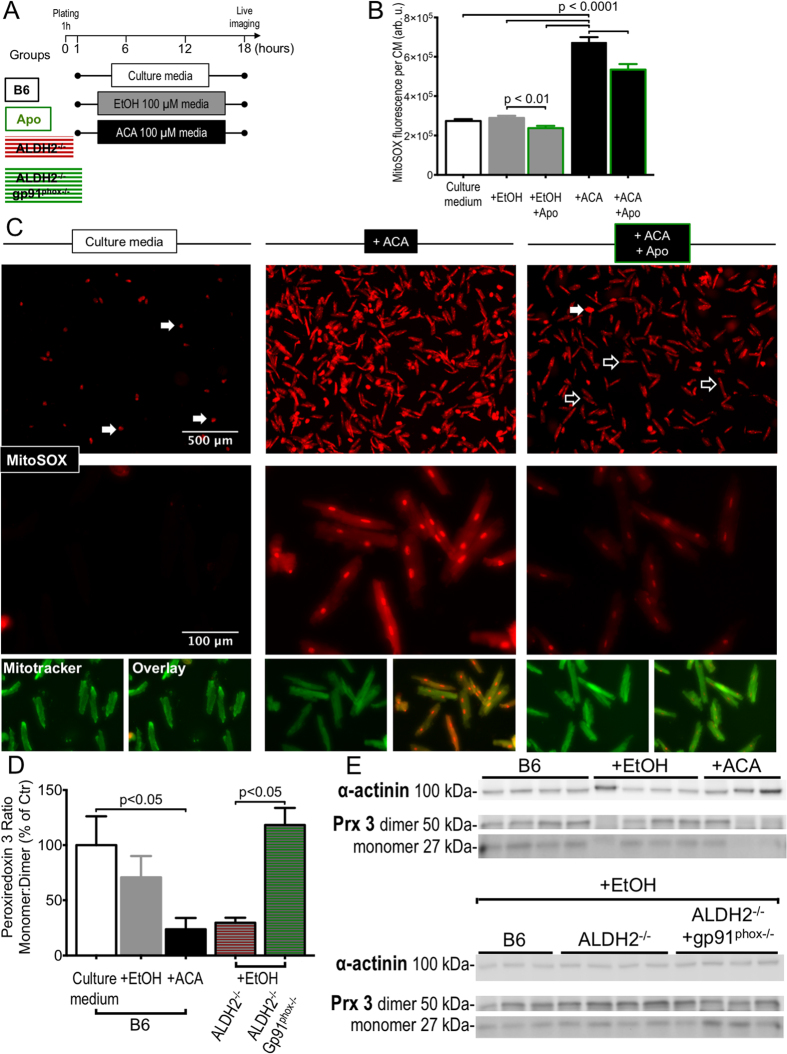
Pharmacologic inhibition or genetic ablation of NOX reduces mitochondrial superoxide production in isolated CMs cultured in the presence of EtOH and ACA. (**A**) Scheme depicting incubation and imaging of isolated adult CMs from B6, ALDH2^−/−^ or ALDH2^−/−^/gp91^phox−/−^ mice with apocynin (300 μM), EtOH (100 μM) or ACA (100 μM). (**B**) MitoSOX-enhanced fluorescence per CMs. Quantification of 205–931 CMs on 6–12 independent low power fields per group. (**C**) Representative fluorescence images of CMs loaded with MitoSOX after culture for 18h in control media (leftmost column) in the presence of ACA 100 μM (middle column) and in the presence of ACA (100 μM) together with the NOX inhibitor apocynin (300 μM) rightmost column. (**D**) Peroxiredoxin 3 ratio monomer/dimer determined by Western blot under non-reducing, denaturing conditions in n** = **4–7 independent samples per group. (E) Representative original Western blots.

To assess whether upregulation of NOX2 subunits and NOX2 activators (Figs 2D–F and 3B,C) and increased NOX activity (Fig. 2C) after EtOH/ACA exposure is associated with increased production of mitochondrial ROS in CMs, as shown in endothelial cells^29^, CMs cultured in the presence of EtOH or ACA with or without the NOX inhibitor apocynin (for the protocol see Fig. 4A) were subjected to automated live cell imaging and image quantification after loading with mitochondrially targeted dihydroethidium-based fluorescent dye MitoSOX (Fig. 4B). CMs cultured under control conditions exhibited low to moderate levels of mitochondrial ROS as long as they persisted in the typical rod-shape, thereby mimicking cells in the intact heart (Fig. 4B,C). Among the control CMs, only the few CMs that had transformed to the “meatball” shape indicating a terminal hyper-contraction displayed increased ROS levels. In contrast, CMs cultured in the presence of ACA showed significantly increased levels of mitochondrial ROS compared to controls. Co-treatment with the NOX inhibitor apocynin significantly reduced mitochondrial ROS levels in ACA-incubated CMs and, to a lesser extent, in EtOH-exposed CMs (Fig. 4B,C, empty arrows: cell in typical rod-shape, filled arrows: cells in terminal hypercontration (“meatballs”)).

MitoSOX, a mitochondrially targeted ROS sensitive fluorescent dye, allows for a quantitative assessment of mitochondrial oxidants within the complex context of live cells, which was required to elucidate the interplay of NOX2 and mitochondria^29^,^30^ in our setting of EtOH/ACA mediated CM dysfunction. However, some inaccuracy as to the exact species, source and location of ROS remains: MitoSOX (Mito-hydroethidine) has multiple oxidation products of which only 2-hydroxyethidium is specific for O_2_^•−^. Also MitoSOX can lead to collapse of ΔΨ_m_ with it or its oxidation products leaking into the cytosol^31^, thereby staining other cell compartments and it has a high affinity for polyanionic macromolecules such as mitochondrial as well as nuclear DNA^32^,^33^. To partially compensate for this (1) we conducted a co-staining of MitoSOX with MitoTracker Green FM, which localizes to mitochondrial membranes irrespective of mitochondrial polarization, and (2) applied an orthogonal assay, the quantification of peroxiredoxin 3, a mitochondrial specific marker for oxidative stress by Western blot^34^,^35^.

As visible in high power images, culture in the presence of ACA resulted in a ROS signal with an even areal distribution throughout the CMs, and which overlapped with the localization of mitochondria as indicated by areal green fluorescence to a large degree. Additional MitoSOX enhanced nuclear fluorescence resulted from the fragility of primary adult CMs with MitoSOX oxidation products leaking from the mitochondria and staining nuclear DNA due to their high affinity for polyanionic macromolecules. Co-treatment with apocynin resulted in an almost complete loss of the mitochondrial MitoSOX signal, underlining the role of NOX2 in the generation of mitochondrial ROS (Fig. 4C).

These data show that pharmacologic inhibition of NOX significantly lowers cytosolic and mitochondrial levels, which were significantly increased after exposure to ACA. Importantly, while the decrease in cytosolic ROS can be explained by a reduced O_2_^•−^ release from NOXs, the reduced levels of mitochondrial ROS hint towards an interplay of NOX activation and mitochondrial ROS production in CMs in our setting of EtOH induced HF/ACA-overload, a mechanism that has been described in angiotensin II-induced oxidative stress^29^,^30^.

Peroxiredoxin 3 (Prx 3, mitochondrial matrix peroxiredoxin) is a mitochondrially located member of the peroxiredoxins, an ubiquitous family of antioxidant enzymes that are involved in cell signaling by regulating H_2_O_2_ levels^36^. Prx 3 exists in a monomeric, reduced thiol form and a dimeric, oxidized disulfide form, while the ratio of reduced to oxidized form serves as a specific marker for mitochondrial oxidative stress as shown by others and by our group^34^,^35^.

Compared to control cultured CMs, CMs cultured in the presence of EtOH or ACA exhibited a gradual reduction of the Prx 3 monomer/dimer ratio, while the ratio in ACA-exposed CMs was significantly reduced as determined by non-reducing Western blot. Of note, CMs isolated from ALDH2^−/−^ exposed to EtOH revealed a monomer/dimer ratio comparable to B6 CMs exposed to equimolar doses of ACA, while the additional lack of gp91^phox^ in CMs isolated from ALDH2^−/−^gp91^phox−/−^ double knockout mice significantly increased the monomer/dimer as compared to EtOH treated ALDH2^−/−^ CMs back to the level of control treated B6 CMs (Fig. 4D,E).

These results further provide an independent support of our hypothesis of increased mitochondrial oxidative stress upon CM exposure to EtOH and especially ACA (directly and indirectly by the lack of ALDH-2) and further stress the role of NOX2 in the induction of mitochondrial ROS.

### Inhibition of NOX attenuates increased mitochondrial ROS formation and improves impaired ΔΨ_m_ in isolated CMs exposed to EtOH/ACA.

In order to test how our results of increased levels of EtOH/ACA induced mitochondrial oxidative stress correlate with mitochondrial ingetrity, we aimed to assess two parameters of mitochondrial function: opening of the mitochondrial permeability transition pore (mPTP) and mitochondrial polarisation (ΔΨ_m_).

**Figure 5:**
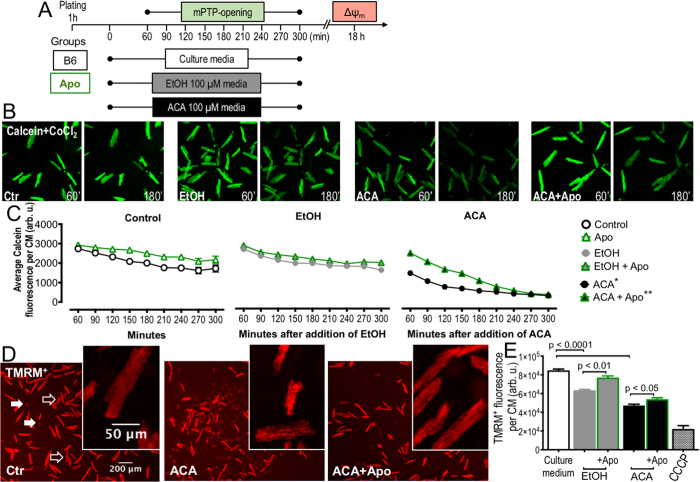
Inhibition of NOX prevents opening of the mitochondrial permeability transition pore (mPTP) and maintains mitochondrial polarization (ΔΨ_m_) in isolated CMs exposed to EtOH/ACA. (**A**) Scheme depicting incubation and imaging of isolated adult CMs with EtOH (100 μM) or ACA (100 μM). (**B**) CM fluorescence after loading with calcein/CoCl_2_ and after incubation with EtOH or ACA and ACA+Apo, representative images. (**C**) Quantification of average calcein/CoCl_2_ derived fluorescence per CM, quantification Calcein/CoCl_2_ enhanced fluorescence of 100 CMs in 6–12 low power fields per group, *p < 0.01 vs Control, **p < 0.01 vs ACA. (**D**) CM fluorescence after loading with the voltage-sensitive fluorescent probe TMRM^+^ and after incubation with ACA and ACA+Apo, representative images. (**E**) Quantification of TMRM^+^-enhanced fluorescence of 416–887 CMs on 6–12 independent low power fields per group, black bar “+CCCP” depicts CM background fluorescence after mitochondrial depolarization with the ionophore/uncoupler carbonyl cyanide *m*-chlorophenyl hydrazone (CCCP) (Fig. S2). (**C,E**) Data presented as the mean ± SEM.

To assess mPTP-opening, CMs cultured in the presence of EtOH or ACA with/without apocynin were loaded with the fluorescent dye calcein-AM, which, after deesterification, is not able to cross the intact inner mitochondrial membrane (IMM) and is trapped in the mitochondria. Further addition of CoCl_2_ quenches only the cytosolic and nuclear fluorescence, as CoCl_2_ cannot enter mitochondria across the IMM (for the protocol see Fig. 5A). While calcein/CoCl_2_ enhanced mitochondrial fluorescence remained stable in control cultured CMs, ACA-cultured CMs exhibited a significant loss of calcein/CoCl_2_ enhanced mitochondrial fluorescence, suggesting a relevant mPTP-opening in these cells. Co-incubation of ACA-exposed CMs with apocynin was able to delay the loss of fluorescence and temporarily prevent mPTP-opening. EtOH-exposed CMs only revealed little loss of fluorescence as compared to control cultured CMs. Interestingly, co-incubation with apocynin seemed to prevent mPTP opening in all three groups, although the effect was most eminent in ACA-cultured CMs (Fig. 5B,C).

To further assess mitochondrial polarization, which represents a key feature of healthy mitochondria and a prerequisite for ATP production by complex V, CMs cultured in the presence of EtOH or ACA with or without apocynin were loaded with the membrane-permeable, voltage-sensitive fluorescent probe TMRM^+^. The majority of control-incubated CMs was rod-shaped and showed bright TMRM^+^-dependent fluorescence, indicating active, polarized mitochondria (Fig. 5D empty arrows), while higher magnification revealed a homogenous distribution of active mitochondria throughout the CM. Only a few CMs with dim fluorescence, indicative of mostly depolarized mitochondria, were visible. Importantly, dim fluorescence and depolarized mitochondria coincided with the terminal hypercontraction, in accordance with to the increased ROS levels (Figs 4C and 5D filled arrows).

Both EtOH- and ACA-cultured CMs showed a significantly reduced TMRM^+^-dependent fluorescence, indicating an overall reduced mitochondrial activity, while at the subcellular level, high magnification revealed a non-homogenously distributed mitochondrial activity. Here, active to hyperpolarized mitochondria were observed adjacent to dark areas devoid of active mitochondria. Many CMs had deteriorated to hypercontraction. Of note, the impact of ACA on mitochondrial activity was more detrimental than EtOH in accordance with the higher ROS levels in these cells (Figs 4C and 5D,E).

Co-treatment with apocynin improved overall mitochondrial activity in both ACA- and EtOH-incubated CMs and reduced the number of hypercontractile CMs. At the subcellular level, it restored the homogenous distribution of active mitochondria (Fig. 5D,E).

The cardinal function of cardiac mitochondria is to generate chemical energy in form of ATP to enable the actin/myosin interaction underlying CM contraction. Our experiments underline the impact of EtOH- and especially ACA-induced, NOX2-mediated mitochondrial ROS on two key parameters of mitochondrial function, namely mPTP-opening and ΔΨ_m_, thereby establishing the link between ACA-overload and CM dysfunction.

### Lack of NOX2/gp91^phox^ protects from HF in a model of ACA-overload.

**Figure 6:**
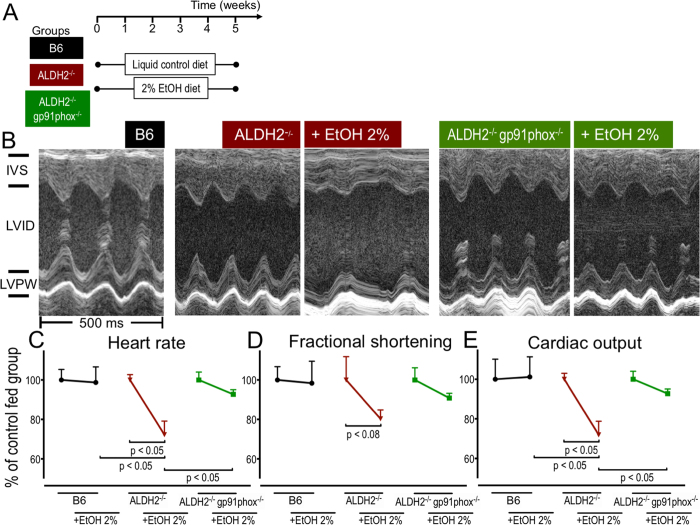
Lack of NOX2/gp91^phox^ protects from alcoholic cardiomyopathy induced by ACA-overload. (**A**) Scheme depicting the treatment of C57BL/6, ALDH-2^−/−^ and ALDH-2^−/−^/gp91^phox−/−^ mice with 2% EtOH diet and liquid control diet. (**B**) Representative M-mode tracings. (**C**) Heart rate, (**D**) Fractional shortening and (**E**) Cardiac output. Data are presented as mean ± SEM from n = 5−12 animals per group and normalized to the respective genotype fed the liquid control diet.

In order to test whether our finding of EtOH/ACA induced, NOX2 mediated increase of mitochondrial ROS leading to impaired mitochondrial and thus impaired CM function, together with the beneficial effects of pharmacologic NOX inhibition or genetic NOX2 ablation *in vitro* could be reproduced *in vivo*, we crossbred ALDH-2^−/−^ mice with mice null for the NOX isoform NOX2 (NOX subunit gp91^phox^) resulting in the ALDH-2^−/−^/gp91^phox−/−^ double knockout mice and subjected mice of all genotypes (C57BL/6, ALDH-2^−/−^ and ALDH-2^−/−^/gp91^phox−/−^ to EtOH vs. control diet (for the protocol see Fig. 6A).

While neither EtOH nor control diet had an effect on cardiac function in C57BL/6 mice, the EtOH diet had a dramatic impact on the cardiac function in ALDH-2^−/−^ mice. Compared to control-fed ALDH-2^−/−^mice, EtOH-fed ALDH-2^−/−^ mice (for the protocol see Fig. 6A) showed a reduced fractional shortening and a significantly decreased heart rate resulting in a significantly impaired cardiac output (Fig. 6B–E). This was consistent with the histomorphometric alterations of cardiac enlargement, pulmonary congestion and increased myocardial fibrosis observed in these animals (Fig. 1B–D).

Additional knockout of NOX2/gp91^phox^ attenuated the detrimental effect of the EtOH diet. It improved fractional shortening and normalized heart rate, resulting in a significantly ameliorated cardiac output in ALDH-2^−/−^/gp91^phox−/−^ double knockout mice as compared to ALDH-2^−/−^ mice (Fig. 6B–E).

### Cardiomyocyte expression of NOX2/gp91^phox^ is increased in patients with HF with reduced ejection fraction and a history of excess alcohol consumption.

**Figure 7:**
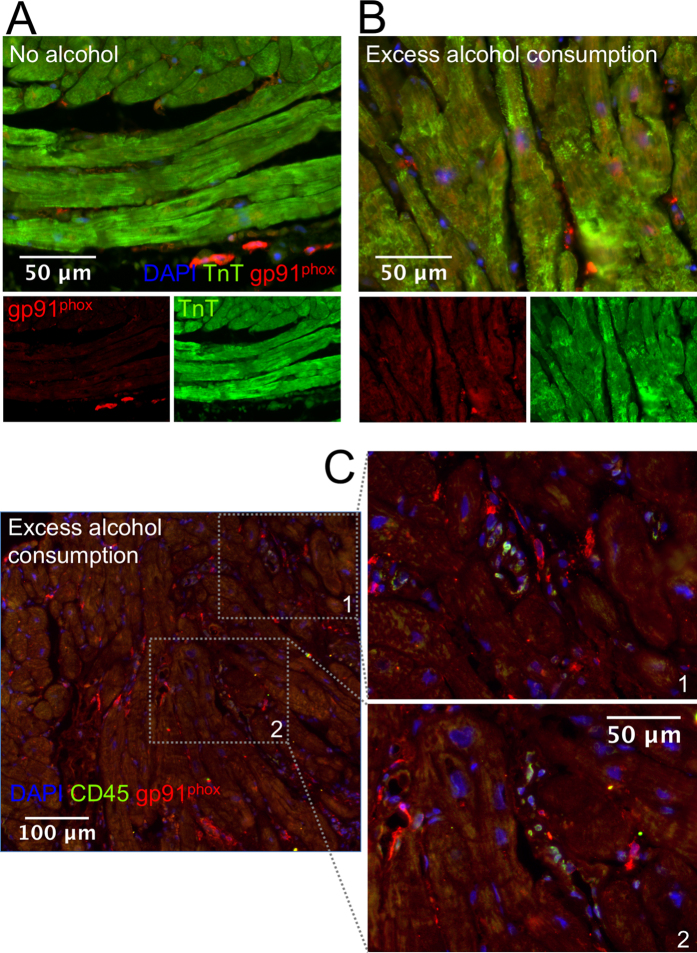
Increased NOX2/gp91^phox^ expression in CMs of HF patients with alcohol abuse. (**A–C**) Representative images from sections from human myocardial biopsies (red, gp91^phox^ staining; blue, DAPI-stained nuclei; A+B green, cardiac troponin T staining; C green, CD45). Excess alcohol consumption was defined as an uptake of EtOH exceeding 60 g per day.

In order to translate our findings to human pathophysiology, we examined patients suffering from HF with reduced ejection fraction who were admitted to our centre for diagnostic work-up. We investigated endomyocardial biopsy specimen taken routinely for diagnostic purposes according to current guidelines. We found a significant upregulation of NOX2/gp91^phox^ expression in CMs from patients with a history of excess alcohol consumption compared to matched controls (Fig. 7 and Table 1). In contrast, cardiac levels of amyloid, incident viral replication (EBV, HHV6, Enterovirus or Adenovirus) or burden of infiltrating CD3^+^ T-cells, Mac-1^+^ myeloid cells or LFA-1^+^ inflammatory cells were not different between groups.

To determine whether the increased myocardial NOX2 expression in our setting of excess alcohol consumption could be due to excessive leukocyte infiltration or due to increased expression of NOX2 by myocardial leukocytes, we conducted a co-staining of the investigated biopsies for NOX2/gp91^phox^ and the leukocyte marker CD45. Interestingly, in biopsies from both groups, the number of myocardial CD45^+^ cells were too low to render a significant contribution of these cells to the myocardial NOX2 expression probable (Fig. 7C).

## Discussion

The onset of ACM correlates with a high daily level of alcohol consumption over an extended period of time^1^. To our knowledge, this is the first study investigating the chronic effects of ACA-overload as a central event in the development of ACM using ALDH-2^−/−^ mice subjected to a long-term EtOH diet. Importantly, the animals displayed key characteristics of ACM^1^, namely the HF phenotype with cardiomegaly and cardiac fibrosis leading to functional cardiac compromise including significant sinus bradycardia, reduced fractional shortening, a significantly reduced cardiac output and consecutive pulmonary congestion, all characteristics that have been described in the human disease phenotype, while these pathologic features were absent in control diet-fed ALDH-2^−/−^ mice and C57BL/6 mice fed the ethanol diet (Fig. 1B–D, Fig. 6B–E). Interestingly, myocardial fibrosis in EtOH-fed ALDH-2^−/−^ mice (Fig. 1D) as an important feature of HF is absent in most murine models of ACM^37^.

We observed a greatly increased susceptibility in ALDH-2^−/−^ mice to EtOH toxicity, which emphasizes the role of ACA in the pathogenesis of ACM. In ALDH-2^−/−^ mice, the phenotype was already apparent after 5 weeks of 2% EtOH liquid diet compared to 14 weeks of 4% EtOH liquid diet in wild type mice reported in earlier studies^13^, while our pilot studies had revealed that ALDH-2^−/−^ mice died after 3 days of 6% EtOH and after 3 weeks of 4% EtOH Liber-diCarli diet feeding.

The ACM phenotype in EtOH-fed ALDH-2^−/−^ mice was accompanied by significantly increased myocardial MDA levels (Fig. 2A,B), which were strongly indicative of increased levels of myocardial ROS as the central phenomenon of ACM in EtOH-fed ALDH-2^−/−^ mice in line with previous studies^6^,^10^. In support of the higher susceptibility of ALDH-2^−/−^ mice for mitochondrial ROS formation, we had previously shown that nitroglycerin treatment results in higher mitochondrial oxidative stress burden in ALDH-2^−/−^ mice as compared to controls^38^. Importantly, the MDA levels correlated with significantly increased NOX activity in myocardial membranous fractions from EtOH-fed ALDH-2 mice compared to control-fed ALDH-2^−/−^ or C57BL/6 mice fed the EtOH-diet (Fig. 2C).

As it is known that ROS play a central role in the pathogenesis of HF, the specific ROS effects depend upon the location, concentration, duration, nature and source of the reactive species. NOX enzymes are capable of ROS production in a tightly regulated fashion. Different NOX isoforms are present in the cardiovascular system, of which NOX2 and NOX4 are the most abundantly expressed isoforms in CMs^22^.

NOX4 seems to comprise cardioprotective abilities, as it was found to protect against chronic pressure overload by counteracting the associated ROS through upregulation of antioxidant genes via Nrf2^39^ and by increasing myocardial angiogenesis via induction of HIF-1α^40^.

The role of NOX2 is more ambiguous: NOX2-derived O_2_^•−^ mediates mechanotransduction and Ca^2+^ signalling in healthy CMs^41^ and in a few specific settings; similar to NOX4, it seems to prevent HF as by decreasing infarct size after preconditioning through improved Ca^2+^ handling in CMs^42^.

However, unlike NOX4, in most contexts NOX2 was also shown to aggravate HF: NOX-2 drives CM hypertrophy in response to stimulation with angiotensin II, noradrenaline, endothelin-1, aldosterone and mechanical stress after aortic constriction through adapter proteins and transcription factors (including Ras/Erk, ASK-1 and NF-κB)^22^. NOX2 is further involved in CM apoptosis via activation of CaMKII^43^. In myocardial fibrosis, NOX2 acts in concert with MMPs, PI3Kγ and CTGF^44^,^45^,^46^.

In line with these previous findings, with our results of significantly increased NOX activity in heart membrane fractions, increased expression of NOX2 activating subunit p67^phox^ and significantly increased expression of NOX2-activating protein Rac1 in myocardial membrane fractions (Fig. 2C–F) and increased expression of NOX2/gp91^phox^, p22^phox^ (NOX2 subunit critical for correct assembly) and Rac1 after culture of isolated CMs in the presence of EtOH/ACA (Fig. 3B,C), we could corroborate previous evidence that NOX2 is upregulated in the development of ACM^13^, while NOX4, the only other NOX isoform expressed in CMs, appeared not to be involved (Fig. S3).

By studying isolated and purified CMs we were able to address the question whether NOX2 is just a bystander or a culprit that exacerbates the myocardial dysfunction in ACM.

Cultured CMs after exposure to ACA exhibited significantly increased levels of cytosolic, and in particular mitochondrial ROS as indicated by MitoSOX-enhanced fluorescence, which overlapped with mitochondrial specific staining to a large degree and as confirmed orthogonally by significant oxidation of the mitochondrial specific enzyme Prx 3^34^,^35^,^36^ in ACA-exposed CMs compared to control cultured CMs (Fig. 4B–E). The increased levels of mitochondrial ROS were paralleled by a significant mPTP-opening and significant reduction of ΔΨ_m_, indicating a compromise of mitochondrial health and cellular function in CMs cultured in the presence of ACA (Fig. 5B–D). Pharmacologic NOX inhibition and genetic ablation of NOX2 significantly reduced mitochondrial ROS (Fig. 4B–E) and were able to significantly delay opening of the mPTP, partially restore ΔΨ_m_ and thereby partially preserve CM mitochondrial function (Fig. 5B–D) and CM morphology (Fig. 3D). These findings hint towards a functional connection of NOX2-derived ROS and mitochondrial dysfunction.

Mitochondrial dysfunction in CMs is a hallmark of HF of different etiologies including ACM^10^,^23^. In our setting, mitochondrial dysfunction mechanistically links excess ROS due to increased NOX2 activity to cardiomyopathy and provides an explanation of how ACA imposes its toxicity on CMs and impairs cardiac function. Using isolated and purified cells, we were able to observe that in CMs, NOX2, which is localized in the cytosol outside the mitochondria, can interfere with intra-mitochondrial processes, presumably using O_2_^•−^ as a signalling molecule in accordance with studies that angiotensin II-induced mitochondrial dysfunction in endothelial cells was shown to be NOX-dependent, as both apocynin and p22^phox^ knockdown were able to preserve mitochondrial function^29^,^30^. Similarly, chronic nitroglycerin treatment induced mitochondrial O_2_^•−^ with subsequent NOX activation leading to the spatially separated phenomena of nitrate tolerance (depending on mitochondrial O_2_^•−^ formation) and endothelial dysfunction (depending on NOX activation)^47^. In this phenomenon, termed “the NOX-mitochondria crosstalk”, NOX2 derived ROS can trigger mitochondrial ROS formation in a protein kinase C, mitochondrial ATP-sensitive potassium channel (K_ATP_ channel) and loss of ΔΨ_m_ dependent fashion as shown by others and our group^29^,^47^,^48^.

The role of NOX2 in the pathogenesis of ACM was further supported by *in vivo* data from mice lacking NOX2/gp91^phox^ in addition to ALDH-2, as these mice were partially protected from the cardiotoxic effects of ACA-overload (Fig. 6).

Furtherly, our findings that NOX2/gp91^phox^ is significantly higher expressed in CMs of individuals with HF and a history of chronic abuse of high amounts of alcohol (>60 g/d) indicate that our experimental findings in principle hold true in human ACM (Table 1, Fig. 7A,B).

While we provide evidence that ACA induces a NOX-2 mediated CM mitochondrial dysfunction in alcoholic cardiomyopathy, it remains to be established how excess ACA leads to increased NOX2 subunit expression and increased NOX activity. The cell cycle regulator protein Cdc42 could provide an explanation: This protein has been shown to mediate the NOX2 activation responsible for oxidative damage induced by EtOH in neurons^28^ (and by arsenic in endothelial cells^27^) and Cdc42 was upregulated in CMs after culture in the presence of EtOH in our hands (Fig. 3C).

Further, the NOX2 activator subunit p67^phox^, which was upregulated in myocardial membrane fractions of EtOH-fed ALDH-2^−/−^ mice, is regulated by the transcription factors PU.1, IRF1 and ICSBP^49^, two of these, PU.1 and IRF1, have been shown to be upregulated by EtOH^50^,^51^ representing a putative molecular mechanism of NOX2 activation in this context.

Finally, as a primary mechanism or in a vicious circle, after initial production of kindling O_2_^•−^-radicals by NOX2, ROS from dysfunctional mitochondria could trigger NOX2 activation in a mPTP-, protein kinase C- and tyrosine kinase cSrc-dependent fashion as recently shown, termed “the mitochondria-NADPH oxidase crosstalk”^52^,^53^.

It remains to be established which ROS source provides the kindling radicals and which one serves as an amplifier of this initial oxidant formation resulting in a vicious circle. In conclusion, our data suggest that myocardial NOX2 in response to ACA overload is involved in the pathogenesis of ACM and interferes with the mitochondrial function in CMs. Pharmacologic inhibition of NOX2, mitochondrial ROS formation and/or mitochondrial permeability might present novel targets to prevent alcoholic cardiomyopathy *in vivo*.

## Material and Methods

### Animal model

All animal experiments were in accordance with the Declaration of Helsinki and National Institutes of Health guidelines. All experiments were approved by the Ethics Committee of the University Hospital Mainz and by the Institutional Animal Care and Use Committee (IACUC; Landesuntersuchungsamt Rheinland-Pfalz, Koblenz, Germany). All mice were housed in a barrier facility (TARC, Translational Animal Research Center, University Medical Center Mainz), kept in filtertop cages with 2–5 mice per cage under specific pathogen-free (SPF) conditions. Male 6–8 weeks old C57BL/6, ALDH-2^−/−^ and ALDH-2^−/−^/gp91^phox−/−^ mice (both on C57BL/6 background) were age-matched and divided into 6 treatment groups comparing control-fed versus EtOH-fed groups of each genotype. The EtOH diet was a modified Lieber-deCarli liquid diet^54^. The 2% EtOH-Lieber-deCarli diet consisted of 20 mL/L 100% EtOH, 61.4 g maltose dextrin and a basal dry mixture, containing casein 41.4 g/L, DL-methionine 0.3 g/L, L-cysteine 0.5 g/L, cellulose 10.0 g/L, xanthan gum 3.0 g/L, corn oil 8.5 g/L, olive oil 28.4 g/L, safflower oil 2.7 g/L, and a standard mineral and vitamin mix for rodent chow (ssniff, Soest, Germany). The control diet consisted of 89.6 g/L maltose dextrin and the basal dry mixture. The mixture was filled up with water to obtain one litre of final solution. Deep-frozen diet was thawed daily and given freshly to the mice. Animals were housed in a 12-hour light-dark-cycle and had free access to the liquid diet, representing the only source of water and nutrition. The treatment protocol was initiated after an accommodation period of 3 days, when animals of all six groups were placed on the liquid control diet. After 5 weeks of treatment, animals were anesthetized by isoflurane inhalation (5% inhalant in room air) and killed by exsanguination via right ventricular puncture. Heart, liver, lung, kidney, spleen and aorta were rapidly excised, transferred to 4 °C Krebs-HEPES-solution (pH 7.35, containing NaCl 99.01 mM, KCl 4.69 mM, CaCl_2_ 2.50 mM, MgSO_4_ 1.20 mM, NaHCO_3_ 25.0 mM, K_2_HPO_4_ 1.03 mM, Na-HEPES 20.0 mM, D-glucose 11.1 mM), cleared of adhesive tissue and snap-frozen in liquid nitrogen.

### Echocardiography

Anaesthesia of mice was induced in a chamber (2–4% isoflurane mixed with 0.2 L/min 100% O_2_) and maintained with a face mask (1–2% isoflurane with 0.2 L/min 100% O_2_). Animals were kept on a heated table mounted on a rail system (Visual Sonics, Toronto, Canada). Ultrasound was performed with the Vevo 770 System and a 40 MHz mouse transducer (VisualSonics). Heart rate was determined. Body temperature was monitored using a rectal probe and maintained at 37 °C. Left ventricular wall thickness, intraventricular septum thickness, left ventricular end-diastolic and end-systolic volumes and left ventricular shortening fraction were determined. Of note, vital parameters including body temperature and anaesthesia were tightly controlled to minimize iatrogenic effects on the murine cardiac physiology.

### Sirius red staining

Hearts were fixed in PFA, embedded in paraffin and sectioned. Paraffin sections were deparaffinized and rehydrated. Nuclei were stained with haematoxylin for 8 min and slides were washed with distilled water (dH_2_O). Slides were then completely covered with Picro-Sirius Red solution (Sirius red F3B, Sigma-Aldrich) 0.5 g in 500 mL of saturated aqueous solution of picric acid (1.3%, Sigma-Aldrich) for 60 min. Slides were washed twice in acidified water (5 mL acetic acid in 1 L of dH_2_O), dehydrated in successive EtOH baths and mounted in synthetic resin. Images were acquired using an Olympus IX71 microscope, Olympus lenses (plan 20x/0.40/oo/0.17 and plan 40x/0.65/oo/0.17) and Olympus colorView U-TV0.5XC-2 camera (Olympus, Tokyo, Japan) at room temperature. Sections of all study arms were analyzed in parallel with identical imaging parameters.

### NOX activity in heart mitochondrial and membrane fractions

Heart mitochondrial and membrane fractions were prepared and measured as previously described^21^,^55^. NOX activity (200 μM NADPH) of the membrane suspensions (0.2 mg/ml protein in PBS) was measured by lucigenin (5 μM)-enhanced chemiluminescence (ECL) after incubation with the substrate NADPH (200 μM). Lucigenin-ECL was quantified in a Lumat LB 9507 luminometer (Berthold Technologies, Bad Wildbad, Germany). Results were normalized for protein content and expressed as counts/mg/min after 5 min.

### Western Blot analysis and dot blot analysis

Heart, lung, liver, kidney and spleen samples were snap-frozen and homogenized in liquid nitrogen. Pulverized tissue was resuspended in ice-cold homogenization buffer (HB, Tris-HCl 20 mM, sucrose 250 mM, EGTA 3 mM, EDTA 20 mM, aprotinin 10 mg/L, leupeptin 5 mg/L, pepstatin 7 mg/L, PMSF 87 mg/L, cantharidin 0.2 mg/L, triton-X 100 1% (v/v)). Adherent, cultured CMs were collected in ice-cold HB. Pulverized tissue/ collected CMs were incubated in HB on ice for 60 min. After centrifugation with 5000 g at 4 °C for 10 min to remove insoluble materials, tissue/CM homogenates were separated by SDS-PAGE (Mini Protean 3, Bio Rad, Hercules, USA) and blotted (Mini-Trans-Blot transfer cell, Bio Rad, Hercules, USA) onto nitrocellulose membranes (Schleicher & Schuell, Dassel, Germany) for Western Blot analysis as described^56^. Briefly, after blocking, immunoblotting was performed with the following antibodies: monoclonal mouse anti α-actinin antibody (1:1000, Cell Signaling, Leiden, Netherlands) as control for loading and transfer and monoclonal mouse anti p67^phox^ antibody (1:1000, Transduction Laboratories, Lexington, USA), mouse monoclonal anti rac1 antibody (1:1000, BD Bioscience, San Jose, USA) and polyclonal goat anti peroxiredoxin 3 antibody (1:340, R&D Systems, Minneapolis, MN, USA). For detection, horseradish peroxidase-labelled secondary antibodies against mouse/rabbit/goat IgG (1:10000, Vector Laboratories, Burlingame, USA) together with ECL reagent (Amersham, Piscataway, USA) were used. The ECL signal was detected with a ChemiLux Imager CsX-1400M (Intas, Göttingen, Germany), while bands were quantified with the Gel-Pro Analyzer software (Media Cybernetics, Bethesda, USA). For dot blot analysis, insoluble materials were removed and tissue homogenates were transferred onto a nitrocellulose membrane with a Minifold I vacuum Dot-Blot system (Schleicher & Schuell, Dassel, Germany) and further processed as described^57^. Briefly, the membrane was washed with PBS and dried at 60 °C for 15 min. For detection of malondialdehyde (MDA)-positive proteins, a polyclonal rabbit antibody against MDA was used (1:2000, Calbiochem, Darmstadt, Germany). Signal detection and quantification was performed as described for Western Blot analysis.

### Isolation, purification and culture of adult murine CMs

Adult murine CMs were isolated as described^58^. Briefly, mouse hearts were digested by retrograde perfusion of type 2 collagenase through the coronary arteries. Live and intact cardiac cells were released by gentle trituration. CMs were purified by repetitive centrifugation due to their characteristic size. After gradual calcium reintroduction, calcium-tolerant cells were plated on mouse laminin-coated dishes in plating media (MEM with Hank’s salts, FBS 10%, 2,3 BDM 10 mM, Penicillin 100 U/mL, glutamine 2 mM and ATP 2 mM). After one hour, the plating medium was removed and cells were washed and switched to culture medium (MEM with Hank’s salts, BSA 0.1%, penicillin 100 U/mL, glutamine 2 mM, insulin 5 μg/mL, transferrin 5 μg/mL, selenium 5 ng/mL and blebbistatin 25 μM). At this stage, only vital and intact CMs were adhering to the plate. CMs were cultured for 18 h in culture medium conditioned with different doses of EtOH or ACA with/without the NOX inhibitor apocynin (300 μM), compared to culture medium alone at 37 °C and 2% CO_2_ in sealed containers to prevent evaporation of EtOH and ACA as described^58^. The incubation time of 18 h was determined empirically as the best compromise of viability of untreated CMs and the most pronounced effect of EtOH/ACA treatment.

### Live imaging of CMs and image quantification

In all steps of culture, loading and imaging, cells were kept at 37 °C and 2% CO_2_. For staining/assessment of CM (1) mitochondrial abundance/mass, (2) mitochondrial O_2_^•−^ production, (3) mitochondrial permeability transition pore (mPTP) opening^59^ and (4) mitochondrial membrane potential (ΔΨ_m_) after exposure to EtOH/ACA, cells were switched to phenol red-free staining medium containing the fluorescent probes: (1) MitoTracker Green FM (100 nM), (2) MitoSOX Red 1 μM, (3) Calcein AM/CoCl_2_ 2 μM/1 mmol, (4) tetramethylrhodamin-methylester (TMRM^+^) 10 nM (all compounds from Molecular Probes / Thermo Fisher Scientific Inc., Darmstadt, Germany) loaded per protocol (1) 15 min, (2) 10 min, (3) 60 min^59^, (4) 30–45 min at 37 °C, 2% CO_2_, in the dark. Cells were imaged in phenol red free culture medium with an automated fluorescence microplate imager with a high-speed laser autofocus and environmental control to minimize phototoxicity due to manual focussing and to obtain unbiased brightness measurements (ImageXpress Micro, Molecular Devices equipped with a Nikon 4 × 0.2 S objective and Semrock filters with the following specifications (Excitation, Emission, Dichroic): TMRM+: 589/15 nm, 632/22 nm, >605 nm and MitoSOX Red: 387/11 nm, 607/36 nm, 580 nm). Images were acquired with the standard 1.4 megapixel cooled CCD after illumination by a 300W xenon light source with the MetaXpress software (Molecular Devices). For some experiments, imaging was conducted with an ArrayScanVTI imaging platform (ThermoFisher Scientific Inc. Berkshire, UK). For each probe, fluorescence of individual CM was quantified after background subtraction to account for uneven illumination by particle analysis using FIJI/ImageJ. To exclude debris, only particles larger than 200 pixels (low power field) were counted. For illustration purposes, monochrome images were pseudo-coloured.

### Real-time quantitative reverse transcription PCR

The mRNA expression was analyzed by quantitative real-time RT-PCR using a 7900HT Fast Real-Time PCR System (Applied Biosystems, Foster City, CA). Briefly, total RNA from CMs aorta was isolated according to the manufacturer’s protocol of the RNeasy mini kit (Qiagen, Hilden, Germany). 50 ng of total RNA was used for real-time RT-PCR analysis with the Power SYBR^®^ Green PCR Master Mix (Thermo Fisher Scientific, Karlsruhe, Germany). Primers sequences were obtained from PrimerBank^60^, Sequences: Rac1 forward: GAGACGGAGCTGTTGGTAAAA, reverse: ATAGGCCCAGATTCACTGGTT. CDC42 forward: CCCATCGGAATATGTACCAACTG, reverse: CCAAGAGTGTATGGCTCTCCAC. GABDH forward: AGGTCGGTGTGAACGGATTTG, reverse: TGTAGACCATGTAGTTGAGGTCA. For the remaining targets, 0.1 μg of total RNA was used for RT-PCR analysis with the QuantiTect Probe RT-PCR kit (Qiagen, Hilden, Germany). TaqMan Gene Expression assays (Applied Biosystems, Foster City, CA) were purchased as probe and primer sets. The comparative Ct method was used for relative mRNA quantification (15). Gene expression was normalized to the endogenous control, TBP or GABPH mRNA, and the amount of target gene mRNA expression in each sample was expressed relative to that of control.

### Human Biomaterial

We analyzed leftover biomaterial from endomyocardial biopsies obtained from patients who were admitted to our center diagnosed with HF with reduced ejection fraction (HFrEF). Endomyocardial biopsies (EMBs) were taken for diagnostic workup of HF of unknown origin according to current treatment guidelines^61^. Written informed consent was obtained from all subjects that leftover biomaterial would be further analysed after pseudonymization. All experiments were in accordance with the Declaration of Helsinki and National Institutes of Health guidelines. All experiments were approved by the Ethics Committee of the University Hospital Mainz.

To assess CM expression of gp91^phox^, we stained myocardial specimen of 6 males with heart failure with reduced ejection fraction (HFrEF) and a history of excess EtOH consumption (e.g., ≥60g EtOH/d) who had unspecific histologic findings in their routinely examined EMBs, and compared them to matched controls without history of alcohol misuse.

### Immunohistochemistry

Slides were deparaffinized in xylene and rehydrated in serial EtOH concentrations. Antigen retrieval was performed in citrate buffer (10 mmol/L sodium citrate, pH 6.0) for 20 min at 99 °C. Slides were blocked with phosphate buffered saline (PBS) with 0.05% Tween-20 and 1% bovine serum albumin (BSA). Primary antibodies were purchased from LifeSpan BioSciences (Seattle, United States) against Nox2/gp91^phox^ (LS-B6247) and from Abcam (Cambridge, United Kingdom) against cardiac Troponin T (ab92546) and CD45 (ab10558). Incubation with primary antibodies was conducted at 4 °C overnight. Secondary antibodies were purchased from Abnova (Heidelberg, Germany) (PAB10815) and from Abcam (ab150116). After three washes in PBS for 5 min, slides were incubated with the secondary antibodies at room temperature in the dark for 1 hour. After three washes in PBS for 5 min each, slides were counterstained with DAPI and mounted. Slides were allowed to cure for 24 hours. Sections were imaged with an Olympus IX73. For quantification, five high power fields were imaged per section. The images were quantified by digital image analysis. To correct for fluorescent background, images were normalized to sections stained with the secondary antibodies only.

### Statistical Analysis

Data are expressed as mean ± SEM. Statistical calculations were performed with GraphPad Prism 5 (GraphPad Software Inc, San Diego, USA). D’Agostino-and-Pearson normality test was first performed, and Kruskal-Wallis test or One-way ANOVA with posthoc Bonferroni multiple comparison test was used as appropriate. Values of p < 0.05 were considered significant.

## Additional Information

**How to cite this article**: Brandt, M. *et al*. NOX2 amplifies acetaldehyde-mediated cardiomyocyte mitochondrial dysfunction in alcoholic cardiomyopathy. *Sci. Rep.* **6**, 32554; doi: 10.1038/srep32554 (2016).

## Supplementary Material

Supplementary Information

## Figures and Tables

**Table 1:** Increased NOX2/gp91^phox^ expression in CMs of HF patients with a history of excess alcohol consumption.

	Dilated cardiomyopathy (DCM)-morphology, no history of alcohol consumption	Dilated cardiomyopathy (DCM)-morphology, with history of excess alcohol consumption	p-Value
N	7	7	
Age (y)	64.3 ± 3.37	62 ± 3.34	p** = **0.3969
Histologic signs of acute or chronic inflammation	0/7	0/7	
History of excess alcohol consumption	0/7	7/7	
Left ventricular ejection fraction (%)	27.14 ± 1.71	24 ± 3.29	p** = **0.7159
LVEDD (cm)	6.66 ± 0.43	6.30 ± 0.26	p** = **0.625
Cardiomyocyte diameter (μm)	19.71 ± 1.51	26.83 ± 2.25	p** = **0.0554
Amyloid	0/7	0/7	
Adenovirus-positive	0/7	0/7	
HHV6-positive	0/7	0/7	
EBV-positive	1/7	0/7	
Enterovirus (Coxsackie)-positive	0/7	0/7	
CD3^+^ cells/mm^2^	3.41 ± 1.31	4.03 ± 2.56	p>0.9999
Mac-1^+^ cells/mm^2^	12.87 ± 2.16	19.98 ± 1.33	p** = **0.0952
LFA-1^+^ cells/mm^2^	10.52 ± 0.92	12.41 ± 4.07	p** = **0.9683
gp91^phox^-Expression	1.35 ± 0.06	1.70 ± 0.06*	**p = 0.0079**

Left ventricular ejection fraction and LVEDD were determined by echocardiography. Histologic and immunohistochemical analyses were performed at the Institute for Cardiac Diagnostics and Therapy (IKDT, Berlin). Gp91phox expression was quantified by digital image analyses after immunostaining. Data are presented as mean ± SEM. HHV6, human herpes virus 6, EBV, Epstein-Barr-virus; Mac-1, integrin α_M_β_2_; LFA-1, lymphocyte function associated antigen 1. Excess alcohol consumption was defined as an uptake of EtOH exceeding 60 g per day.
