# Supplementary material for: NOX2 amplifies acetaldehyde-mediated cardiomyocyte mitochondrial dysfunction in alcoholic cardiomyopathy
Source: Sci Rep. 2016 Sep 14;6:32554. doi: 10.1038/srep32554 (PMC5021994; doi:10.1038/srep32554)
Supplement: Supplementary Information [file srep32554-s1.pdf]

Supplementary data

NOX2 amplifies acetaldehyde-mediated cardiomyocyte mitochondrial dysfunction in alcoholic cardiomyopathy

Moritz Brandt[1,2], Venkata Garlapati[2], Matthias Oelze[1], Efthymios Sotiriou[1], Maike Knorr[1,2], Swenja Kröller-Schön[1], Sabine Kossmann[1], Tanja Schönfelder[2], Henning Morawietz[4], Eberhard Schulz[1,3], Heinz-Peter Schultheiss[5], Andreas Daiber[1,3], Thomas Münzel[1,2,3], Philip Wenzel[1,2,3]

From the [1]Center for Cardiology, Cardiology 1, [2]Center for Thrombosis and Hemostasis Mainz and German [3]Center for Cardiovascular Research (DZHK), partner site Rhine Main, University Medical Center Mainz, Langenbeckstr. 1, 55131 Mainz; [4]Division of Vascular Endothelium and Microcirculation, Department of Medicine III, University Hospital Carl Gustav Carus, Technische Universität Dresden, Fetscherstr. 74, 01307 Dresden, Germany, [5]Institut für kardiale Diagnostik und Therapie, Moltkestrasse 31, 12203 Berlin, Germany

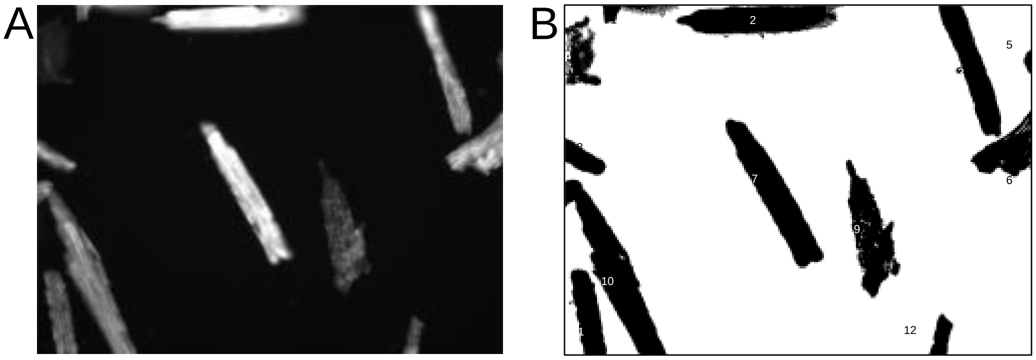

C

CM number	Area	Mean	StdDev	Min	Max	IntDen	RawIntDen
1	4600	255.387	59.767	139	458	1.174.778.000	1.174.778.000
2	32880	445.305	177.027	142	907	14.641.614.000	14.641.614.000
3	27265	309.368	99.411	140	589	8.434.905.000	8.434.905.000
4	12220	166.863	12.934	137	229	2.039.066.000	2.039.066.000
5	1042	192.710	32.281	140	293	200.804.000	200.804.000
6	17857	346.850	89.078	141	600	6.193.694.000	6.193.694.000
7	38404	396.916	135.940	141	796	15.243.144.000	15.243.144.000
8	7340	264.667	84.382	139	480	1.942.655.000	1.942.655.000
9	30471	211.515	41.641	138	435	6.445.083.000	6.445.083.000
10	46644	279.717	64.633	138	491	13.047.110.000	13.047.110.000
11	16962	243.283	43.758	136	370	4.126.574.000	4.126.574.000
12	5208	217.780	52.671	136	417	1.134.196.000	1.134.196.000

Figure S1: Image quantification to measure MitoSOX- or TMRM$^+$-enhanced fluorescence per cardiomyocyte. After background subtraction to account for uneven illumination, raw images (A) were subjected to particle analysis algorithms (B) yielding multiple parameters per CM (C).

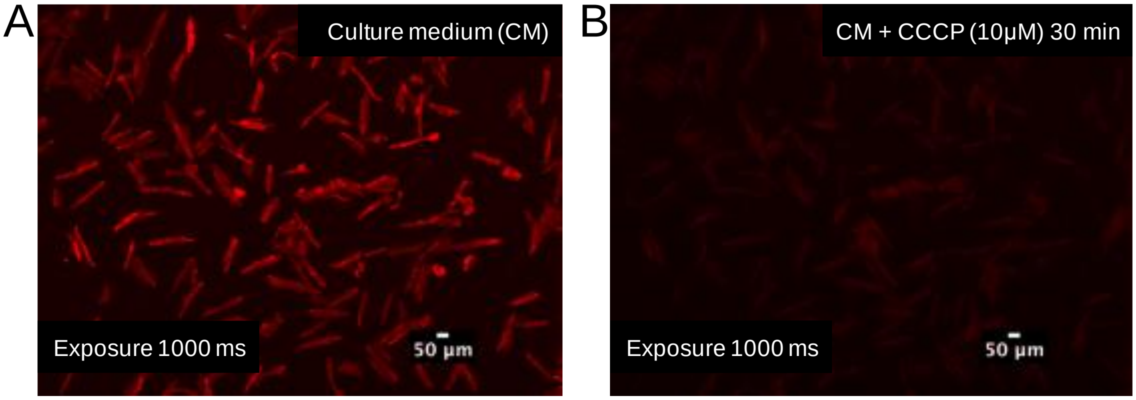

Figure S2: Addition of the ionophore/mitochondrial uncoupler carbonyl cyanide *m*-chlorophenyl hydrazone (CCCP) to CM loaded with TMRM$^+$ (A) resulted in an almost complete loss of TMRM$^+$-enhanced fluorescence indicating a complete mitochondrial depolarization (B).

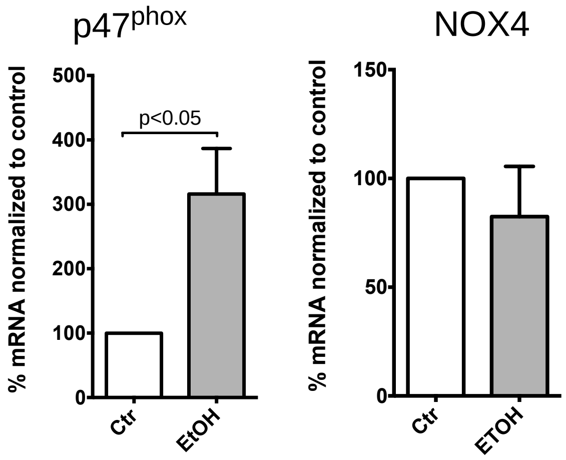

Figure S3: Effect of ethanol exposure of isolated cardiomyocytes on the expression of the NADPH subunit p47^{phox} and NADPH oxidase 4 (NOX4). Data presented as the mean±SEM.

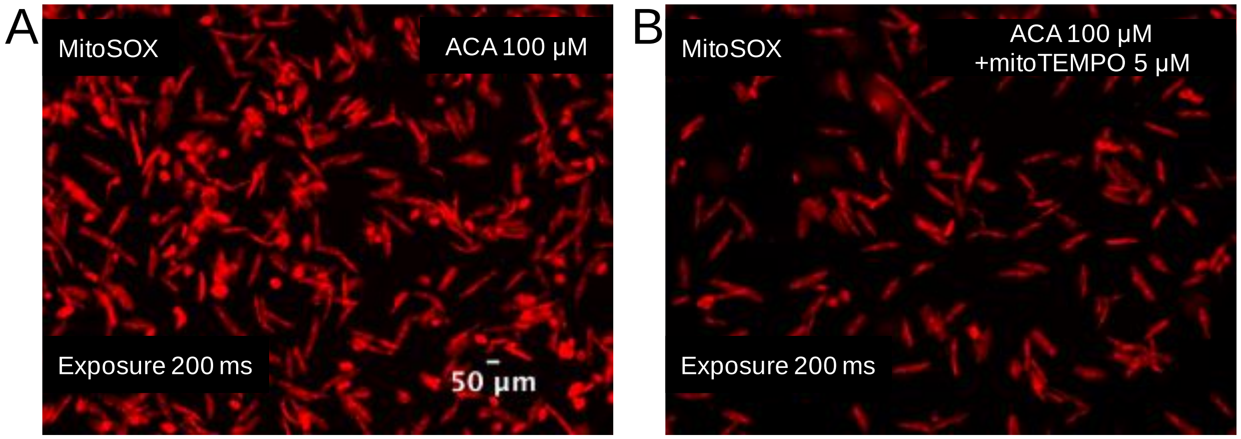

Figure S4: Effect of the mitochondrial specific antioxidant mitoTEMPO on CM ROS after exposure in culture media containing ACA 100 µM.
